# South West Orthopaedic Club

**Published:** 1989-11

**Authors:** 


### South West Orthopaedic Club

### Meeting at the Royal Gwent Hospital, Newport on November 12th 1988

### "OUR EXPERIENCES OVER TEN YEARS WITH 500 KNEE LIGAMENT RE-CONSTRUCTIONS FOR ANTERO-LATERAL AND COMBINATION INSTABILITIES, USING NORMAL TISSUE AND NO SYNTHETIC MATERIAL"

**I. G. Mackie, MB FRCS**
Cardiff Royal Infirmary
Newport Road, Cardiff
**Mervyn J. Cross, MB FRACS**
North Sidney Sports Injury Centre,
Ridge Street, Sidney, NSW

Our experiences started over twelve years ago initially using semi tendonosis and gracilis as a transarticular substitute for the anterior cruciate (ACL) with Mackintosh extra-articular support, followed by six weeks immobilisation in an above knee plaster cast, followed by intensive physiotherapy. The results of this initial procedure were very good, however, we have modified our technique to, on occasions, substituting the ACL with patellar tendon providing accurate ligament placement with isometric guide. We now use an extra-articular dynamic lateral support which protects the repair before mobilisation of the knee, using a cast brace with the final 40° of extension blocked for the first six weeks. The results from the development of this technique show 85%–90% excellent results with the patients returning to their initial activities at international sport level, and the remaining 10% improved over their pre-operative state, but not to the level they desire.

### ANTERIOR CRUCIATE LIGAMENT REPAIR—POSTOPERATIVE ASSESSMENT OF FOUR METHODS

**E. J. Smith, J. G. Hussell, J. H. Newman**

Bristol Royal Infirmary

Anterior cruciate ligament (ACL) rupture results in instability of the knee joint. A process of "patient selection" takes place and this is usually followed by ACL repair.

The results of forty patients with ACL laxity repaired by four different methods, Insall, MacIntosh, Jones (modified), and synthetic ligament implants were compared. A functional score, activity grading, and stability testing as well as measurement with a KT1000 arthrometer were obtained for each patient.

One patient (3%) was able to maintain the equivalent level of sport, 11 patients (28%) were unable to pursue any activity, while the remaining 69% performed at a lower level.

Sixteen patients (40%) [four MacIntosh, five Jones, one Insall, six synthetic ligament repairs] were regarded as having an excellent or good result. In sixteen patients (40%) [eight MacIntosh, three Jones, three Insall, two synthetic ligament repairs] the operative results were deemed fair. In two (5%) patients results were poor and in another six (15%) the operations failed (two MacIntosh, two Jones, three Insall, one synthetic ligament repair]. Five of these patients suffered from instability and/or pain and swelling of the knee joint.

The mean functional scores suggest that the Insall repair does not give as good a result as do the other operative groups.

### SPHERICAL SILASTIC SPACER ARTHROPLASTY OF THE FIRST METATARSOPHALANGEAL JOINT

**Peter May, Mika Togantzi, Nickolas Granitzas, John Kirkup**
Bath and Wessex Orthopaedic Hospital, Bath

There is still no clear consensus over the best treatment for the painful valgus, or the stiff first metatarsophalangeal joint. Surgeons have applied the principals of replacement arthroplasty inserting, for example, large finger joints (Whalley, 1975). In 1982 Helal published the results of forty two patients treated by replacement of the first MTP joint with a spherical silastic spacer: in Bath the senior author (JRK) has used this device since 1981.

We briefly review the operative technique in the light of this experience and illustrate some important features.

We present the results of a retrospective review of all those patients whose records indicated replacement surgery of the first MTP joint between 1981 and 1987. Fifty four patients were identified and sent initial questionaires before recall for review and x-ray. Pre-operative records have been analysed for details of pain, walking ability and joint movement; valgus has been measured on routine pre-operative standing radiographs.

At review the range of passive movement was measured. (A neutral posture for the first MTP joint is difficult to define depending on medial arch changes with rest or weightbearing.

In this unit range is measured relative to the *axis of the metarsal* shaft. This will be discussed as it alters values of dorsiflexion or plantar flexion quoted alone although the "total arc" of movement can be compared with other reports.)

Forty three of these patients have received a Helal's silastic spherical spacer for either painful, degenerate hallux valgus or hallux rigidus. Eleven other patients had been treated with Swanson's prostheses and were similarly reviewed as a separate cohort. Of the Helal group, sixteen patients had undergone bilateral procedures; after exclusion for inadequate follow-up, there were *fifty one joints* for analysis. Age at operation ranged from twenty nine–seventy six years, median fifty five years. At review ALL remained mobile and independant; eighty five percent of the patients were very pleased with their result although some had taken up to one year to settle.

Five joints had been removed, mostly within the first year. Although on culture one grew staph aureus, four were sterile but exhibited a florid fibrous response, suggesting possible reaction to the material.

Grading of results for such surgery should be based on patient satisfaction, comfort and mobility and we shall discuss our classification by four grades "Excellent" to "Poor". Fourteen percent of joints in our review could be graded "Excellent" and sixty eight percent—"Good" (comfortable but with <fifty percent of range).

The Arc of movement found in these joints ranged from 0° to 75°, median 25°.

Because of the neutral point chosen, fifty percent of joints reviewed had no actual plantar flexion although they did have 15°–20° of plantar movement from the standing position. Only two patients had fixed dorsiflexion deformities.

Eighty two percent of this series had Excellent or Good results. All but seven patients were very pleased with the result of their operation. Ability to be mobile with comfortable feet is of greater value than an excessive range of movement.

### EARLY RESULTS OF CHIELECTOMY AS TREATMENT FOR HALLUX RIGIDUS

**H. Benbarka and G. Jones**
Royal Gwent Hospital, Newport, South Wales

Prospective study done at The Royal Gwent Hospital. 12 chielectomies in 11 patients and results assessed.

Procedure (Chielectomy) Dorsal incision centred over metatarsophalangeal joint. Incise capsule—Underscore dorsal, medial and latal aspect of metatarsal head. Excise dorsal osteophyte along with one quarter of metarsal head, the osteophyte at base of proximal phalanx, and the bunion if present.

Close in layers, wool and crepe. Post op. Heel Walking with passive and active excercises. Discharge in 1–3 days.

Patients were assessed for pain and movement. Results—Pain—8 pain free, 3 mild pain, 1 moderate to severe. Movement. All had increased movement with dorsiflexion of 30–40 degrees. Patients avoid wearing high heels more than 2 inches. Complications, nil.

Conclusion. Chielectomy is a simple procedure with satisfactory early results. We recommend it as initial treatment for Hallux Rigidus, if it fails the option of arthrodesis or silastic implant is still feasible.

### HANSSON PINS FOR HIP FRACTURES

**N. R. Clay, S. R. Johnson, S. L. Lewis**
The Cardiff Royal Infirmary

Hansson Pin fixation has been employed for all subcapital hip fractures in Cardiff for over one year. The development of a percutaneous pinning technique under regional anaesthesia has reduced physical and physiological trauma to a minimum.

One hundred and sixty five patients (83% Garden III or IV fractures) have entered this prospective trial, and preliminary results after an average seven month follow-up are presented. 8.5% of patients have died since operation. 90% of surviving patients were mobile at review—one third without aid—and 77% had mild or no pain. 77% were living independently or in supervised accommodation. Failure of fixation has occurred in 17.5% of cases, mostly within three months of operation.

All fixation failures were in Garden III and IV fractures and were more common with less experienced surgeons, although this was not statistically significant. two subtrochanteric fractures have occurred at the site of pin insertion. Pin trip irritation of muscles was common, especially after fracture site impaction. These patients will be reviewed again two years post-operatively.

### THE INFLUENCE OF WRIST POSITION ON THE MINIMUM FORCE REQUIRED FOR ACTIVE MOVEMENT OF THE INTERPHALANGEAL JOINTS

**R. Savage**
Cardiff Royal Infirmary

Following flexor tendon repair, techniques that protect the repair whilst healing occurs include passive mobilisation and controlled dynamic mobilisation. Splintage with the wrist *flexed* is thought to reduce tension in the flexor tendons. But if *active* mobilisation is to be used, is this the correct position for splinting the wrist?

Active and passive muscle tension is discussed in relation to finger flexor and extensor tendons. Minimising *active* tension required to produce finger movement is considered to be an important part of post-operative mobilisation following flexor tendon repair, in which active movement is used. It is argued that "minimal active tension" in the flexors is equal to, or just exceeds, the passive tension in the extensors. A method of measuring passive tension in finger tendons has been described. In twenty-four volunteers, it has been used to determine that if the meta-carpophalangeal joints are held flexed, there is least "minimal active tension" in the flexor tendons when the wrist is splinted in *extension*.

### PITFALLS IN THE USE OF INTERLOCKING NAILS FOR FEMORAL FRACTURES

**R. Majkowski and A. S. Baker**
Bristol Royal Infirmary

Previous reports highlight the potential of the interlocking nail in the management of femoral fractures. This clinical and radiological review of the first 41 cases performed in a general orthopaedic and trauma unit identifies three particular problem areas.

The patients ranged between fifteen years and seventy eight years of age (mean thirty eight years) at the time of their injuries. The internal fixations were performed up to eleven days after injury (mean 4.8 days). Static fixation was used in nineteen femurs. Eight of these were subsequently dynamized between eight and twenty six weeks (mean 18.6 weeks) post operatively. Fractures were located in the proximal third of the femoral shaft in twelve cases, in the middle third in sixteen and in the distal third in thirteen. The fractures were grouped by their comminution index (Winquist 1984). Eleven were Type I, seven Type II, seven Type III, one Type IV, four were segmental and two were nailed prophylactically. *Nine were not* known at the time of this submission.

The insertion point of the nail was particularly important in proximal third fractures. Of the twelve femurs with proximal third fractures the nail was inserted via the greater trochanter in four. All had low varus malalignment, ranging from 3°–10° (mean 7°) and *two had comminution of the fracture site*. Of the eight fractures where the nail was inserted via the piriform fossa, there was no malalignment. *The trochanteric insertion point was also associated with varus malalignment of three out of six middle third fractures* but not in distal third fractures.

Valgus malalignment occurred in five of the thirteen distal third fractures ranging from 6° to 12° (mean 8.8°). Shortening of more than 1 cm occurred in four of these fractures, three of which had been fixed in the dynamic mode. These four were oblique or spiral fractures.

Six patients were osteoporotic, five had valgus malalignment between 7° and 12° (mean 7.5°) despite supplementary fixation in three cases with cerclage wires or Partridge bands.

This study demonstrates the importance of using the piriform fossa as the insertion point, especially for the more proximal fractures. Valgus malalignment is likely when fractures of the distal third are nailed with patients in the latral position. It is therefore recommended that for distal third fractures either nailing is performed with the patient supine, or with an obliquely placed lower femoral traction pin, running from distomedial to proximolateral. When the lower third fractures are oblique or spiral the static mode should be used to prevent shortening. It is also suggested that strong supplementary fixation is usually required for osteoporotic fractures if malunion is to be avoided.

This report identifies pitfalls in the use of interlocking nails. This study is particularly important now that the use of these devices is moving from the specialist centres to general orthopaedic and trauma units.

### THE TREATMENT OF SUBTROCHANTERIC FRACTURES OF THE FEMUR USING THE DYNAMIC HIP SCREW

**J. M. Britton, C. M. L. H. Gibbons, R. A. Al Mufti and P. C. May**
Bath and Wessex Orthopaedic Hospital, Royal United Hospital, Bath

In previously reported series of subtrochanteric fractures treated by reduction and nail-plate fixation, the rate of implant failure has been between 17 and 40%. Since 1981, we have used the Dynamic Hip Screw (DHS) in preference to other devices because of its apparently improved design.

Between 1981 and 1986, 80 patients with a subtrochanteric fracture were treated using a DHS. Patients under the age of 60 with high velocity fractures and those with pathological fractures were excluded leaving 55 patients (48 female, 7 male: average age 80.5 years) in the series.

Using the method devised by the American Society of Anesthesiologists (1978), patients were assessed for pre-existing systemic disease. In 28% there was none. In 44% it was mild, in 23% severe but not incapacitating and in 5% severe and incapacitating. Their level of dependence was classified by Hall and Ainscow's system (1981) as fully independent (63%), sheltered (32%) or institutionalised (5%).

The fractures were classified by Seinsheimer's method (1978) as undisplaced (2%), 2-part (28%), 3-part (28%), 4-part (12%) or subtrochanteric with intertrochanteric extension (30%).

Reduction and fixation of each fracture was carried out in the standard manner. In 37 patients a four or six-hole plate was used: 18 had plates of eight, ten or twelve holes. The mean operating time was 77 minutes and the average intraoperative blood loss 510 mls. Each patient spent an average of 5 days in bed postoperatively before walking with a frame and was discharged 12 days later.

Fourteen patients (24.6%) died within 3 months of operation. Their mean age was 86 years. In each case the combination of fracture and operation was thought to be a major contributory factor. Each had pre-existing systemic disease and in 8 cases this was severe. Only 5 of the 41 surviving patients (12.2%) failed to return to their previous level of independence. A sheltered existence and severe pre-existing disease both predisposed to death within three months. Early death was unrelated to fracture type, grade of operating surgeon, plate length, duration of operation and blood loss.

The fixation failed on three occasions (5%). In two cases the lag screw cut out of the femoral neck due to poor placement; in one case the lower screws pulled out of the femur. This figure is significantly lower than those previously reported using other devices. There were no other major differences between the series.

We conclude that the Dynamic Hip Screw provides considerably better fixation of subtrochanteric fractures than other nail-plates. The high mortality rate is related to age, pre-existing disease and increasing social dependence but is unaffected by the method of fixation.

### EXTERNAL FIXATOR OR REMANIPULATION FOR UNSATISFACTORY COLLES REDUCTION?

**C. B. D. Lavy, R. Woodwards**
Royal United Hospital, Bath

We report a radiological review of 50 patients (aged 16 to 80) with fractures of the distal radius who underwent apparently satisfactory closed reduction, but on subsequent X-ray (2 to 14 days later) were found to have slipped to an unacceptable position.

25 were further treated by remanipulation and 25 with an external fixation device. The two groups were matched for age, sex and degree of displacement.

Review of X-rays at 6 to 12 weeks following the fracture shows improved correction of radial length, radial deviation and dorsal tilt in the group treated with external fixation.

